# Emission and biosynthesis of volatile terpenoids from the plasmodial slime mold *Physarum polycephalum*

**DOI:** 10.3762/bjoc.15.281

**Published:** 2019-11-28

**Authors:** Xinlu Chen, Tobias G Köllner, Wangdan Xiong, Guo Wei, Feng Chen

**Affiliations:** 1 Department of Plant Sciences, University of Tennessee, Knoxville, TN 37996, USA; 2 Department of Biochemistry, Max Planck Institute for Chemical Ecology, Hans-Knöll-Strasse 8, D-07745 Jena, Germany

**Keywords:** amoebae, evolution, terpene synthases, volatiles

## Abstract

Terpene synthases (TPSs) are pivotal enzymes for the production of diverse terpenes, including monoterpenes, sesquiterpenes, and diterpenes. In our recent studies, dictyostelid social amoebae, also known as cellular slime molds, were found to contain *TPS* genes for making volatile terpenes. For comparison, here we investigated *Physarum polycephalum*, a plasmodial slime mold also known as acellular amoeba. Plasmodia of *P. polycephalum* grown on agar plates were found to release a mixture of volatile terpenoids consisting of four major sesquiterpenes (α-muurolene, (*E*)-β-caryophyllene, two unidentified sesquiterpenoids) and the monoterpene linalool. There were no qualitative differences in terpenoid composition at two stages of young plasmodia. To understand terpene biosynthesis, we analyzed the transcriptome and genome sequences of *P. polycephalum* and identified four *TPS* genes designated *PpolyTPS1*–*PpolyTPS4*. They share 28–73% of sequence identities. Full-length cDNAs for the four *TPS* genes were cloned and expressed in *Escherichia coli* to produce recombinant proteins, which were tested for sesquiterpene synthase and monoterpene synthase activities. While neither PpolyTPS2 nor PpolyTPS3 was active, PpolyTPS1 and PpolyTPS4 were able to produce sesquiterpenes and monoterpenes from the respective substrates farnesyl diphosphate and geranyl diphosphate. By comparing the volatile profile of *P. polycephalum* plasmodia and the in vitro products of PpolyTPS1 and PpolyTPS4, it was concluded that most sesquiterpenoids emitted from *P. polycephalum* were attributed to PpolyTPS4. Phylogenetic analysis placed the four *PpolyTPSs* genes into two groups: *PpolyTPS1* and *PpolyTPS4* being one group that was clustered with the TPSs from the dictyostelid social amoeba and *PpolyTPS2* and *PpolyTPS3* being the other group that showed closer relatedness to bacterial TPSs. The biological role of the volatile terpenoids produced by the plasmodia of *P. polycephalum* is discussed.

## Introduction

Volatile organic compounds (VOCs) are used by many living organisms as chemical languages for communication [1–2]. Rapid progress has been made in our understanding of the VOC world of microbes, especially bacteria [3–4] and fungi [5–6]. Not only the chemical diversity of microbial VOCs is continuingly to be discovered, our understanding of their biosynthesis is also growing rapidly [7–8]. Among the diverse VOCs, terpenoids are the largest group. Terpenoids are biosynthesized from two C5 diphosphate compounds isopentenyl diphosphate (IPP) and its isomer dimethylallyl diphosphate (DMAPP), which are produced by either the mevalonate (MVA) pathway or the methylerythritol phosphate (MEP) pathway [9–10]. The MVA pathway is found in eukaryotes, archaea, and a few bacteria, and the MEP pathway is present in several photosynthetic eukaryotes and bacteria [11]. Isoprenyl diphosphate synthases (IDSs) catalyze the formation of prenyl diphosphates of various chain length [12]. After that, terpene synthases (TPSs) catalyze the conversion of prenyl diphosphates to diverse terpenes [13]. Because all living organisms produce prenyl diphosphates, whether an organism has the ability to produce terpenes depends on whether it contains *TPS* genes.

Recently we could show that dictyostelid social amoebae contain *TPS* genes. *TPS* genes were found in 6 species of sequenced amoeba, including *Dictyostelium discoideum*, *D. purpureum, Cavenderia fasciculata* (formerly *D. fasciculatum*), *Tieghemostelium lacteum* (formerly *D. lacteum*), *Heterostelium album* (formerly *Polysphondylium pallidum*), and *Actyostelium subglobosum* [14]. The number of *TPS* genes ranges from 1 to 21 in these species. Some of the *TPS* genes among these species have conserved catalytic functions. For example, TPSs of one orthologous group that include DdTPS6, DpTPS1, AsTPS1, DiTPS1, DfTPS1, and PpTPS18 all catalyze the formation of the sesquiterpene protoillud-7-ene [15–16]. Among paralogs, there is dramatic functional divergence. For instance, *D. discoideum* contains 9 *TPS* genes with diverse catalytic activities [14]. In *D. discoideum*, most *TPS* genes showed expression during multicellular development [14–15]. Consistent with the catalytic activities and gene expression patterns, the products of most DdTPSs were released as volatiles from *D. discoideum* at the multicellular developmental stage [14–15].

*TPS* genes previously were known to exist only in bacteria, fungi, and plants [13,17–18]. The identification of *TPS* genes in dictyostelid social amoeba now indicates a broader distribution of *TPS* genes. To understand whether *TPS* genes occur in other groups of amoebae, in this study, we investigated *Physarum polycephalum*. *P. polycephalum* belongs to the class of Myxogastria whereas social amoeba belongs to the class of Dictyostelia, but they both belong to the same infraphylum Mycetozoa. *P. polycephalum* is called plasmodial amoeba because of the plasmodium formed during the vegetative phase. Plasmodium is a single cell containing millions of nuclei, which also gives the name of acellular amoeba. *P. polycephalum* has been a popular model organism for studying a diversity of topics [19–21], ranging from cytoplasmic streaming to primitive intelligence [22]. In a manuscript deposited at arXiv [23], it was described that complex mixtures of volatiles including some terpenoids were detected from the headspace of *P. polycephalum* using two extraction temperatures. In our study, we aimed I) to determine whether *P. polycephalum* releases volatile terpenoids under normal growing conditions and II) to identify and characterize the genes for terpene biosynthesis in *P. polycephalum*. Our results will enable us to compare terpene chemistry and their underpinning biosynthetic genes in the two lineages of amoeba.

## Results

### Plasmodia of *P. polycephalum* release a mixture of volatile terpenoids

**Figure 1:**
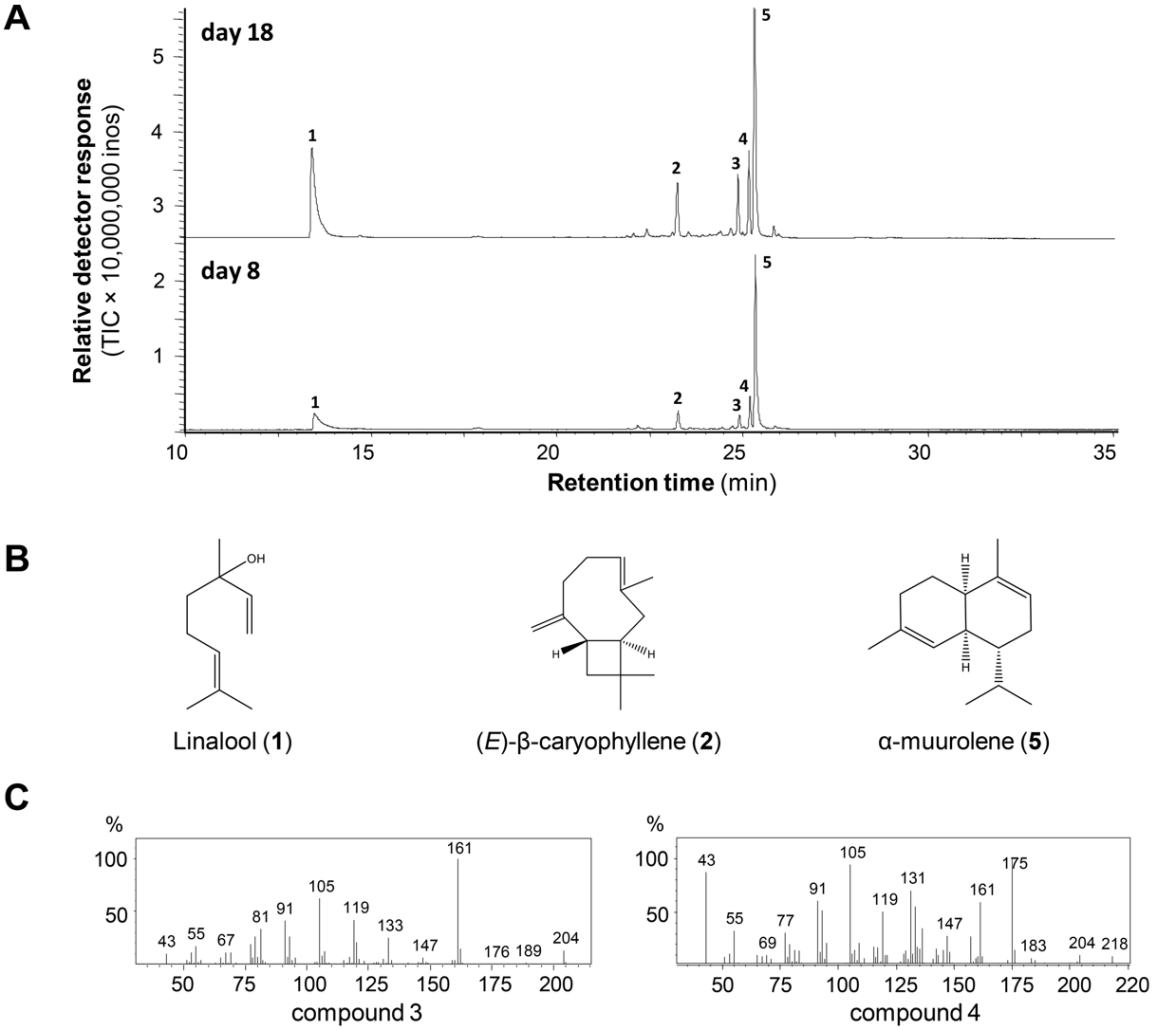
Plasmodia of *P. polycephalum* emit a mixture of volatiles predominated by terpenoids. A) GC chromatograms of volatiles collected from plasmodia of *P. polycephalum* grown at 8 and 18 days after transferring to a fresh agar plate. This experiment was repeated three times with similar results. **1**, linalool; **2**, (*E*)-β-caryophyllene; **3**, unidentified sesquiterpene hydrocarbon (compound **3**); **4**, unidentified putative sesquiterpene aldehyde (compound **4**); **5**, α-muurolene. B) Structures of known terpenoids. The structure of compound **2** shows the (−)-enantiomer of (*E*)-β-caryophyllene. However, since we have not performed chiral analysis, the absolute configuration of (*E*)-β-caryophyllene emitted from *P. polycephalum* plasmodia is still unknown. C) Mass spectra of unidentified terpenoids.

To determine whether *P. polycephalum* releases volatile terpenoids like dictyostelid social amoebae, plasmodia of *P. polycephalum* were cultured on agar plates with oak flakes as nutrient source and subjected to volatile profiling at two time points: 8 days and 18 days after the transfer of plasmodia to a fresh agar plate. Volatiles were collected from the headspace using a solid phase-microextraction fiber and analyzed using gas chromatography–mass spectrometry (GC–MS). At the 8th day after the transfer, a total of five volatiles were detected, including three known compounds and two unidentified compounds (Figure 1A). The three known compounds are all terpenoids, including one monoterpene linalool and two sesquiterpenes (*E*)-β-caryophyllene and α-muurolene (Figure 1B). The two unidentified are putative sesquiterpenoids. Compound **3** is a putative hydrocarbon sesquiterpene with a molecular mass of 204 (Figure 1C). In contrast, compound **4** has a molecular formula of C_15_H_22_O and a molecular mass of 218 (Figure 1C). It was predicated to be a sesquiterpene aldehyde. At the 18th day after the transfer, essentially the same profile of volatiles was detected (Figure 1A).

### Four terpene synthase genes were identified in *P. polycephalum*

**Figure 2:**
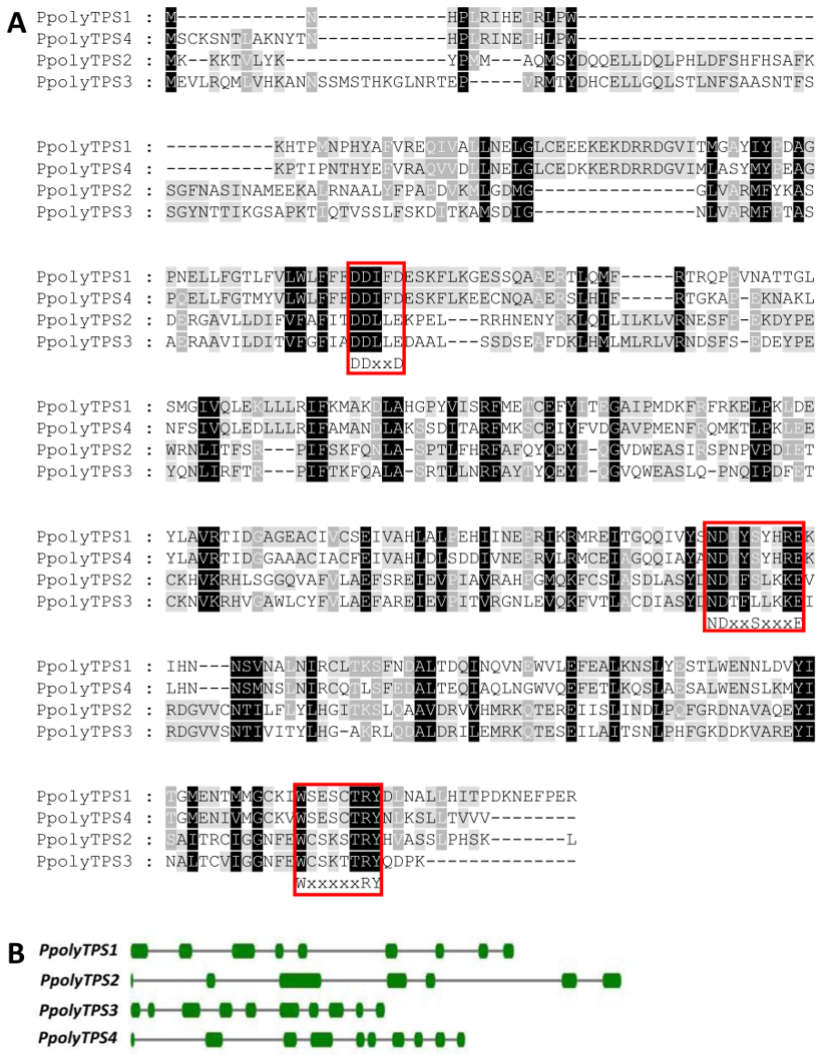
*P. polycephalum* contains four terpene synthase genes. A) Multiple sequence alignment of the protein sequences of the four PpolyTPSs. The sequences for PpolyTPS1-PpolyTPS4 reported in this paper have been deposited in the GenBank database (accession numbers. MN523652–MN523655). Three signature motifs for terpene synthases were boxed. Shadings in black and gray indicate identical and similar residues, respectively. B) Intron/exon organization of the four *PpolyTPS* genes. Boxes and lines indicate exons and introns, respectively.

With the identification of terpenes from the headspace of *P. polycephalum* (Figure 1), the next question was how they are synthesized. The genome of *P. polycephalum* has been sequenced [24] and there are multiple transcriptome datasets available for this species (http://www.physarum-blast.ovgu.de). Because the genome sequence was not annotated, we searched the transcriptomes for *TPS* genes. A total of four full-length putative *TPS* genes were identified from the transcriptomes. They were designated as *PpolyTPS1*, *PpolyTPS2*, *PpolyTPS3,* and *PpolyTPS4*. The length of the proteins encoded by *PpolyTPS1*, *PpolyTPS2*, *PpolyTPS3,* and *PpolyTPS4s* is 334, 347, 353, and 337 amino acids, respectively. Among the four proteins, the highest sequence similarities occurred between PpolyTPS1 and PpolyTPS4 (72%) and between PpolyTPS2 and PpolyTPS3 (64%). PpolyTPS1/4 and PpolyTPS2/3, however, showed only ≈30% sequence similarity to each other. Terpene synthases can be classified into class I and class II, based on the reaction mechanisms they catalyze. These two types of terpene synthases are associated with conserved motifs: class I TPSs contain a ‘DDxxD/E’ and a ‘NSD/DTE’ motif while class II TPSs contain a ‘DxDD’ motif. While PpolyTPS1 and PpolyTPS4 contain the ‘DDxxD’ motif, PpolyTPS2 and PployTPS3 contain a ‘DDxxE’ motif. PpolyTPS1, PpolyTPS4, and PpolyTPS2 contain the ‘NDxxSxxxE’ motif. This motif is changed to “NDxxLxxxE” in PpolyTPS3. Based on their motifs, all four PpolyTPSs can be predicted to be class I TPSs. Also observed in all four PpolyTPSs is the “WxxxxxRY” motif (Figure 2A), which is frequently found in TPSs [25]. In our analysis of the genome sequence of *P. polycephalum*, the coding sequences of all four *PpolyTPS* genes were identified. *PpolyTPS* genes contain six to nine introns (Figure 2B). This is in contrast to the *TPS* genes from dictyostelid social amoeba, which consist zero to three introns [14].

### Biochemical activities of PpolyTPSs

To determine whether *PpolyTPS* genes encode functional terpene synthases, full-length cDNAs were amplified by RT-PCR and cloned into the protein expression vector pEXP-5-CT/TOPO.

**Figure 3:**
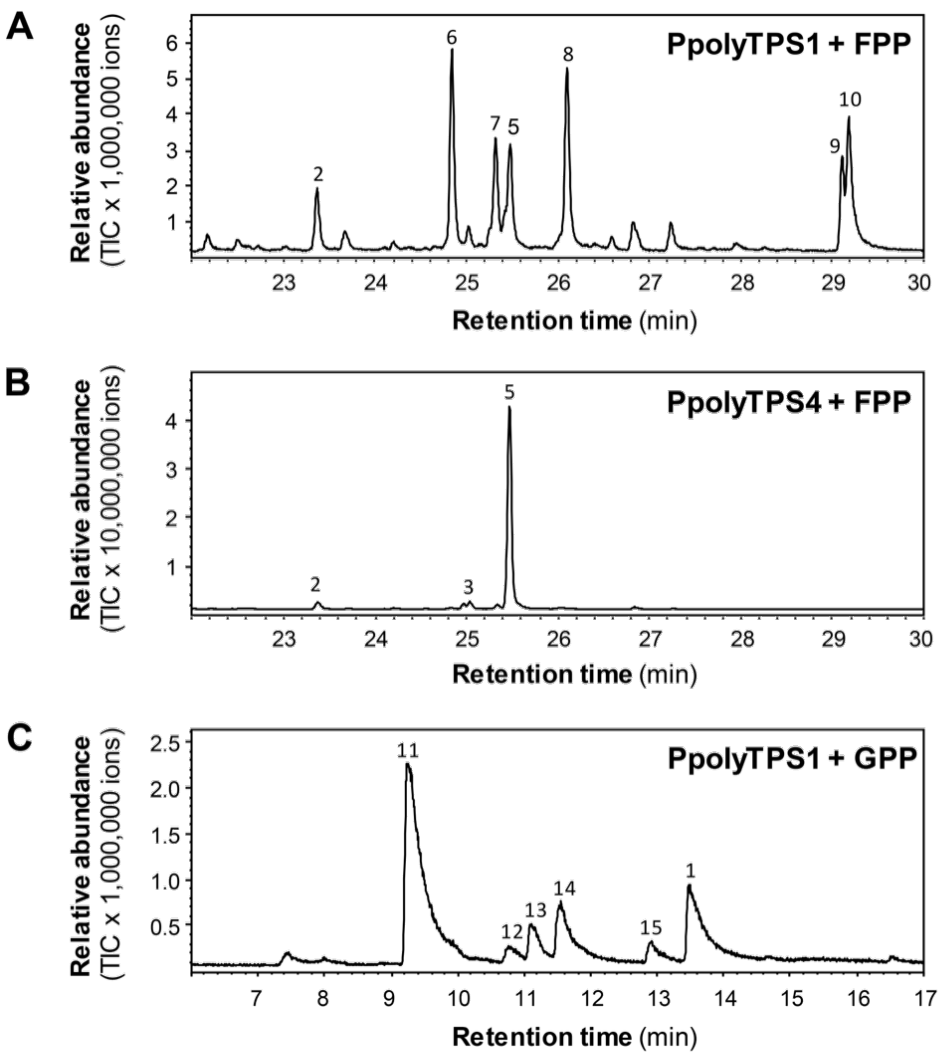
PpolyTPS1 and PpolyTPS4 have terpene synthase activities. A) GC chromatogram of sesquiterpenes produced by recombinant PpolyTPS1 incubated with FPP. **2**, (*E*)-β-caryophyllene; **6**, γ-muurolene; **7**, unidentified sesquiterpene 1; **5**, α-muurolene; **8**, unidentified sesquiterpene 2; **9**, unidentified sesquiterpene 4; **10**, unidentified sesquiterpene 5. B) GC chromatogram of sesquiterpenes produced by recombinant PpolyTPS4 incubated with FPP. **2**, (*E*)-β-caryophyllene; **3**, unidentified sesquiterpene 3; **5**, α-muurolene. C) GC chromatogram monoterpenes produced by recombinant PoplyTPS1 incubated with GPP. **1**, linalool; **11**, myrcene; **12**, limonene; **13**, (*Z*)-β-ocimene; **14**, (*E*)-β-ocimene.

Recombinant PpolyTPSs were heterologously expressed in *Escherichia coli* and then tested for terpene synthase activities using geranyl diphosphate (GPP) and (*E,E*)-farnesyl diphosphate (FPP) as substrates. PpolyTPS2 and PpolyTPS3 did not show detectable terpene products with either GPP or FPP. In contrast, PpolyTPS1 could convert GPP into a mixture of cyclic and acyclic monoterpenes, including myrcene and linalool (**1**, Figure 3). PpolyTPS4 showed only trace activity with GPP and produced very small amounts of myrcene. When using FPP as substrate, PpolyTPS1 produced a mixture of sesquiterpenes with γ-muurolene as the most abundant compound and (*E*)-β-caryophyllene (**2**), α-muurolene (**5**), and four unidentified sesquiterpenes (Supporting Information File 1, Figure S1) as minor components. PpolyTPS4 converted FPP into α-muurolene, (*E*)-β-caryophyllene, and one unidentified sesquiterpene (Figure 3). As negative controls, neither denatured PpolyTPS1 nor denatured PpolyTPS4 produced any terpene products using FPP (Supporting Information File 1, Figure S2).

### Relatedness of PpolyTPSs to the TPSs from dictyostelid social amoebae, fungi, and bacteria

**Figure 4:**
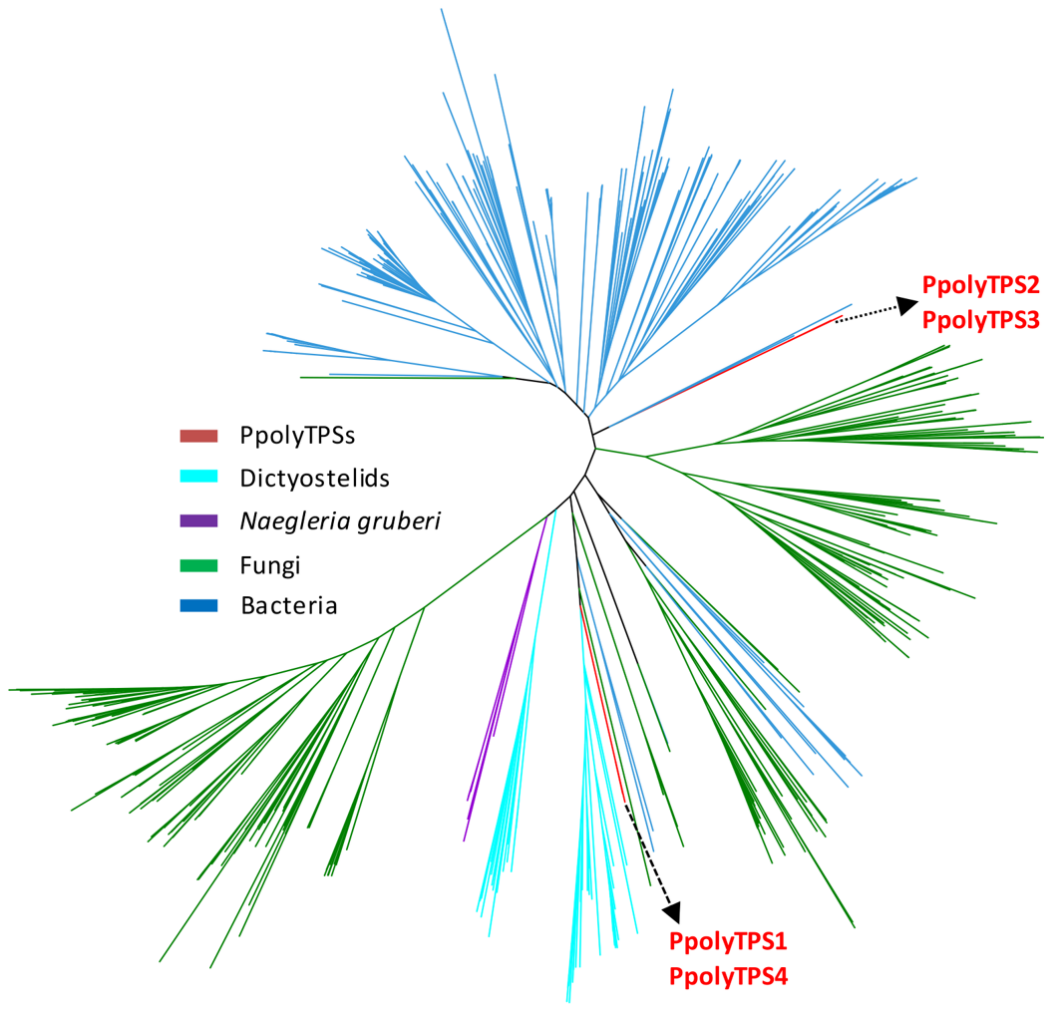
Phylogenetic analysis of PpolyTPSs with TPSs from dictyostelid social amoebae (Dictyostelids), the amoebae *Naegleria gruberi*, fungi, and bacteria. TPSs from dictyostelid social amoebae were obtained from the study [15]. TPSs from another amoeba *N. gruberi*, fungi, and bacteria were obtained from the study [14]. TPSs from different lineages were color-coded.

When individual *PpolyTPS* genes were used as query to search against the nonredundant protein database at NCBI, the top hits for PpolyTPS2 and PpolyTPS3 were all from bacteria. In contrast, the top hits for both PpolyTPS1 and PpolyTPS4 were from eukaryotes (Supporting Information File 1, Table S1). To further understand the evolutionary relatedness of PpolyTPSs to other TPSs, we performed a phylogenetic analysis of PpolyTPSs with TPSs from dictyostelid social amoeba, another amoeba *Naegleria gruberi*, fungi, and bacteria. The four PpolyTPSs genes were divided into two groups (Figure 4). *PpolyTPS1* and *PpolyTPS4* formed one group that was clustered with the TPSs from the dictyostelid social amoeba and the amoeba *N. gruberi*, which together were more related to fungal TPSs. In contrast, *PpolyTPS2* and *PpolyTPS3* formed the other group that showed closer relatedness to bacterial TPSs.

## Discussion

With our previous work, now we have shown that both the dictyostelid social amoebae [14–15] and *Physarum polycephalum* (Figure 1A) release mixtures of volatiles predominated by terpenoids. Despite the fact that both are amoeba, dictyostelids and *P. polycephalum* have a long evolutionary distance and exhibit strikingly different life styles. In social amoeba, volatile terpenoids are exclusively released at the multicellular stage and were not detected from the unicellular vegetative stage [15]. In contrast, volatile terpenoids from *P. polycephalum* were released from plasmodia, its vegetative stage. Interestingly, the volatile terpenoids from both types of amoeba are predominated by sesquiterpenoids. However, α-muurolene, the most abundant sesquiterpene released from *P. polycephalum*, was not found in *D. discoideum* and *D. purpureum*. There were differences between the volatiles we detected in this study and the ones detected from *P. polycephalum* in a previous study [23]. In the Kateb and Costello study [23], a total of 87 compounds and 79 compounds were identified at the incubation temperature of 75 °C and 30 °C, respectively, from the plasmodia of *P. polycephalum.* Besides the differences in temperature (75 °C is not biologically relevant), the plasmodia were extracted from the agar plate before headspace collection in the Kateb and Costello study [23], which would disrupt the culture. Therefore, the much larger number of compounds detected in that study may be partly due to the destructive nature of their method. It is notable that α-muurolene (**5**) was detected from both studies.

Like in social amoeba, the production of most volatile terpenoids in *P. polycephalum* can be attributed to specific terpene synthases. PpolyTPS4 produced α-muurolene (**5**) as a major product, and (*E*)-β-caryophyllene (**2**) and an unidentified sesquiterpene hydrocarbon as minor products. These three terpenes could also be found in the headspace of *P. polycephalum* with α-muurolene as the most abundant constituent (Figure 1A). It is thus conceivable that PpolyTPS4 is responsible for the formation of these three compounds in vivo. PpolyTPS1 produced also α-muurolene, however, the in vitro product profile was dominated by other sesquiterpenes that were not detected in the headspace of *P. polycephalum*. Nonetheless, PpolyTPS1 possessed monoterpene synthase activity in vitro and produced a mixture of monoterpenes. One of the PpolyTPS1 monoterpene products, linalool (**1**), could be detected in the headspace of *P. polycephalum*, but the other monoterpene products including myrcene, limonene, (*Z*)-β-ocimene, and (*E*)-β-ocimene were not detected from the headspace of *P. polycephalum*. It is thus unclear whether and how PpolyTPS1 contributes to volatile terpene formation in the vegetative stage of *P. polycephalum*. It is possible that *P. polycephalum* contains other *TPS* genes, which were not identified in this study due to incomplete transcriptome and genome information, but contribute to the in vivo biosynthesis of linalool. It is interesting to note that only PpolyTPS1 and PpolyTPS4 are closely related to TPSs of dictyostelid social amoebae whereas PpolyTPS2 and PpolyTPS3 are closely related to bacteria TPSs (Figure 4), suggesting two evolutionary origins of *PpolyTPS* genes. It is tempting to speculate that *PpolyTPS2* and *PpolyTPS3* may be derived from bacteria through horizontal gene transfer, which was recently demonstrated to have occurred from bacteria to fungi for the evolution of *TPS* genes [26]. It is also interesting to note that under our standard assay conditions, neither PpolyTPS2 nor PpolyTPS3 showed activity with either GPP or FPP. Because catalytic motifs are present in both TPSs (Figure 2), the inactivity is somehow puzzling. Some efforts are needed to discern whether they are instable enzymes, active with different prenyl diphosphate substrates, or require different conditions for catalysis. It is also possible that they become inactive genes in the process of pseudogenization.

What are the biological functions of volatile terpenoids emitted from *P. polycephalum*? In plants and other organisms, volatile terpenoids have many biological/ecological functions. They may serve as chemical defense [27–28] or as signals for attracting beneficial organisms. For social amoeba, multiple functions have been proposed, including chemical defense, attracting spore dispersers, and regulating development. In *D. discoideum*, a mutant strain with a disrupted *DdTPS8* gene showed slower progression in development [29], adding genetic evidence on the role of *TPS* genes and their terpene products in development. The volatiles emitted from *P. polycephalum* may have similar functions as those in dictyostelid social amoeba or have lineage-specific functions. *P. polycephalum* has a unique biology. It sends information in the form of nutrient concentrations through the tubular network using the streaming cytoplasm [30]. It will be interesting to test whether volatile terpenoids function in internal communication of *P. polycephalum*. When foraging, *P. polycephalum* marks the territory that have been explored. Some chemicals have been proposed to function in this process [31]. It is well known that some insects such as fire ants use terpenes as trace pheromone [32]. It will be interesting to determine whether some terpenoids produced by *P. polycephalum* have similar functions*.* When studying chemotaxis of *P. polycephalum*, a number of exogenously applied volatiles were tested for attraction and repellence. One sesquiterpene farnesene was found to be a strong chemoattractant of *P. polycephalum* [31]. Thus, it will also be intriguing to ask whether volatile terpenoids emitted from *P. polycephalum* are involved in chemotaxis.

## Conclusion

In this study, we have successfully identified and characterized terpene synthase (*TPS*) genes that are involved in making volatile terpenoids from a plasmodial slime mold *Physarum polycephalum*. The volatiles emitted from the plasmodium of *P. polycephalum* were mainly sesquiterpenes, but also included monoterpenes (Figure 1). These volatile terpenoids are probably constitutively produced, because the plasmodia at two developmental stages of *P. polycephalum* did not display qualitative differences in terpene profiles (Figure 1). Four *TPS* genes, designated *PpolyTPS1*–*PpolyTPS4*, were identified from *P. polycephalum*. When *E. coli*-expressed PpolyTPS proteins were tested for terpene synthase activities, PpolyTPS1 was demonstrated to be a sesquiterpene synthase and PpolyTPS4 a monoterpene synthase (Figure 3). Their in vitro terpene products were also detected from the headspace of *P. polycephalum* plasmodium. When PpolyTPSs were compared with those from dictyostelid social amoebae, only *PpolyTPS1* and *PpolyTPS4* were shown to be clustered with the TPSs from the dictyostelid social amoeba (Figure 4). Despite this difference as well as the difference in life style of *P. polycephalum* and dictyostelid social amoeba, it appears that both of these two types of organisms use volatile terpenoids for certain biological and ecological processes. This study provides novel information on the occurrence of terpenoids and their biosynthetic genes among eukaryotes. Because *P. polycephalum* is a popular model system for investigating many aspects of biology and other disciplines such as engineering and physics [19,22,33–36], our results most likely will stimulate new research directions using *P. polycephalum* as a model system centered upon volatile terpenoids.

## Experimental

### *P. polycephalum* culture

*P. polycephalum* was purchased from Carolina Biological Supply Company (https://www.carolina.com). The plasmodium of *P. polycephalum* was cultured on agar plates with oat flakes as a nutrient source under continuous darkness.

### Headspace collection and GC–MS analysis

On the 8th day and 18th day after the transfer of plasmodia to a fresh agar plate, volatiles were collected from the headspace of plasmodia on the agar plate using solid phase microextraction (SPME) (https://www.sigmaaldrich.com). After collection for one hour, the SPME fiber was withdrawn and then inserted into the injector port of a Shimadzu 17A gas chromatograph coupled to a Shimadzu QP5050A quadrupole mass selective detector for volatile identification and identification. Separation was performed on a Restek Rxi-5Sil MS column (30 m × 0.25 mm i.d. × 0.25 µm thickness; Restek) with helium as the carrier gas and a temperature program from 60 °C to 300 °C at 5 °C per minute rate. The experiment was performed with three biological replicates.

### Sequence retrieval and analysis

Transcriptome datasets for *P. polycephalum* were downloaded from the *Physarum polycephalum* Genome Resources database (http://www.physarum-blast.ovgu.de). Protein sequences were predicated using TransDecoder (5.0.2) [37]. Putative terpene genes were searched against SmMTPSLs HMM profile [38] using HMMER 3.0 hmmsearch [39] with an E-value of 1e^−5^. The coding region of each *PpolyTPS* gene was identified from the genome sequence of *P. polycephalum*. For phylogenetic reconstruction, a multiple sequence alignment was first performed using MAFFT (v7.450) [40] in accurate strategy (L-INS-i) with 1000 iteration of improvement. Then phylogenetic trees were built by Fasttree [41] and visualized using interactive tree of life (ITOL) (https://itol.embl.de/).

### Cloning of full-length cDNA of terpene synthase genes in *P. polycephalum*

Plasmodia were harvested from agar plates, placed in 2 mL centrifuge tubes and disrupted using Qiagen TissueLyser II according to manufacturer’s manual (https://www.qiagen.com). Total RNA was isolated using Plant RNA Purification Reagent (https://www.thermofisher.com). cDNA was prepared using 1st strand cDNA synthesis kit (https://www.gelifesciences.com). Full length cDNAs of individual *PpolyTPS* genes were amplified using gene specific primers (Table S2, Supporting Information File 1), cloned into pEXP5 CT/TOPO vector (https://www.thermofisher.com) and fully sequenced.

### Terpene synthase enzyme assays

pEXP5 CT/TOPO vector containing individual *PpolyTPS* genes was transformed into *E. coli* strain BL21 Codon Plus (DE3). Heterologous expression of individual *PpolyTPS* genes in *E. coli* and recombinant protein preparation and terpene synthase enzyme assays were performed as previously described [42]. Each PoplyTPS recombinant protein was tested with both geranyl diphosphate and farnesyl diphosphate as substrates and terpene products were analyzed using GC–MS as described for volatile profiling.

## Supporting Information

File 1 Additional figures and tables.
