# Supplementary material for: Emission and biosynthesis of volatile terpenoids from the plasmodial slime mold Physarum polycephalum
Source: Beilstein J Org Chem. 2019 Nov 28;15:2872–80. doi: 10.3762/bjoc.15.281 (PMC6902781; doi:10.3762/bjoc.15.281)
Supplement: File 1 — Additional figures and tables. [file Beilstein_J_Org_Chem-15-2872-s001.pdf]

BEILSTEIN JOURNAL OF ORGANIC CHEMISTRY

Supporting Information

for

Emission and biosynthesis of volatile terpenoids from the plasmodial slime mold *Physarum polycephalum*

Xinlu Chen, Tobias G. Köllner, Wangdan Xiong, Guo Wei and Feng Chen

Beilstein J. Org. Chem. **2019,** *15,* 2872–2880. doi:10.3762/bjoc.15.281

Additional figures and tables

License and Terms: This is a supporting information file under the terms of the Creative Commons Attribution License (http://creativecommons.org/licenses/by/4.0). Please note that the reuse, redistribution and reproduction in particular requires that the authors and source are credited.
The license is subject to the *Beilstein Journal of Organic Chemistry* terms and conditions: (https://www.beilstein-journals.org/bjoc)

Table S1. List of top 5 gene hits for each PpolyTPS as query against the NCBI Non-redundant protein sequence database

Query	Top hits	species	% identity	evalue	Lineage
	KIK52964.1	*Gymnopus luxurians* FD-317 M1	27.239	2.33E-18	Eukaryota
	XP_001836556.1	*Coprinopsis cinerea* okayama7#130	28.782	4.02E-18	Eukaryota
	KIK91194.1	*Paxillus rubicundulus* Ve08.2h10	26.282	5.64E-18	Eukaryota
	WP_073739522.1	*Streptomyces* sp. CB02488	26.299	3.56E-17	Bacteria
PpolyTPS1	WP_143140611.1	*Nannocystis exedens*	25.083	6.08E-17	Bacteria
	SER09932.1	*Nitrosomonas* sp. Nm51	28.364	1.99E-23	Bacteria
	WP_094328294.1	*Nostoc* sp.	26.266	2.62E-22	Bacteria
	WP_017313508.1	*Fischerella* sp. PCC 9339	26.129	7.16E-22	Bacteria
	WP_131117423.1	*Westiellopsis* prolifica	26.129	7.53E-22	Bacteria
PpolyTPS2	WP_135107638.1	*Mastigocladus laminosus*	26.129	1.12E-21	Bacteria
	WP_017313508.1	*Fischerella* sp. PCC 9339	25.095	4.27E-18	Bacteria
	WP_135107638.1	*Mastigocladus laminosus*	25.095	4.57E-18	Bacteria
	WP_131117423.1	*Westiellopsis prolifica*	24.735	1.19E-17	Bacteria
	WP_026736531.1	*Fischerella* sp. PCC 9605	25.095	4.36E-17	Bacteria
PpolyTPS3	WP_096675321.1	*Fischerella* sp. NIES-4106	24.335	3.47E-16	Bacteria
	KIK91194.1	*Paxillus rubicundulus* Ve08.2h10	27.108	2.49E-18	Eukaryota
	KIJ62846.1	*Hydnomerulius pinastri* MD-312	26.377	3.62E-18	Eukaryota
	WP_143140611.1	*Nannocystis exedens*	25.298	1.68E-17	Bacteria
	XP_638489.1	*Dictyostelium discoideum* AX4	24.096	1.80E-17	Eukaryota
PpolyTPS4	KIO00339.1	*Pisolithus tinctorius* Marx 270	26.298	2.68E-17	Eukaryota

Table S2. Primers used for cloning of full-length cDNAs of *PpolyTPS* genes.

Gene name	Primers	Sequences(5'-3')
PpolyTPS1	Forward	ATGAATCACCCTCTACGCATCCACGAAATC
	Reverse	CTAACGCTCGGGAAATTCATTTTTATC
PpolyTPS2	Forward	ATGAAAAAAAAGACTGTCTTGTATAAATATC
	Reverse	TTAAAGTTTTGAGTGGGGAAGGCTG
PpolyTPS3	Forward	ATGGAGGTTTTGAGACAAATGCTG
	Reverse	TCACTTGGGGTCTTGGTATCTGGTC
PpolyTPS4	Forward	ATGTCCTGCAAAAGCAATACTCTCG
	Reverse	CTATACTACCACAGTCAAAAGTGATTTC

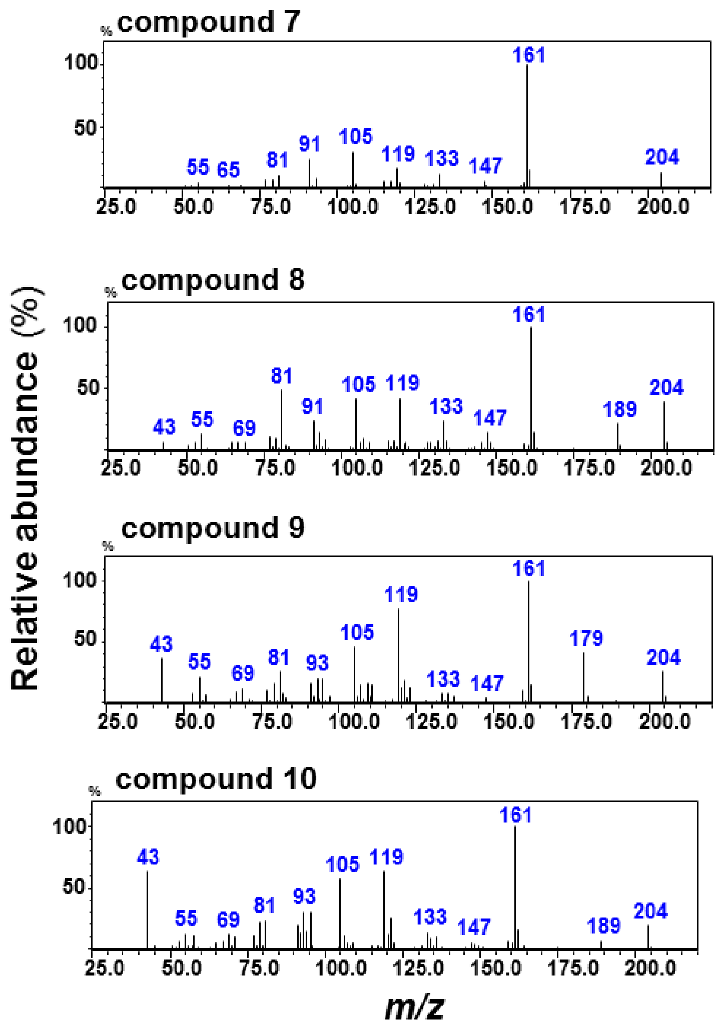

Figure S1. The mass spectra of unknown sesquiterpenes described in Fig. 3. The numbering of the different sesquiterpenes follows the nomenclature given in Fig.3.

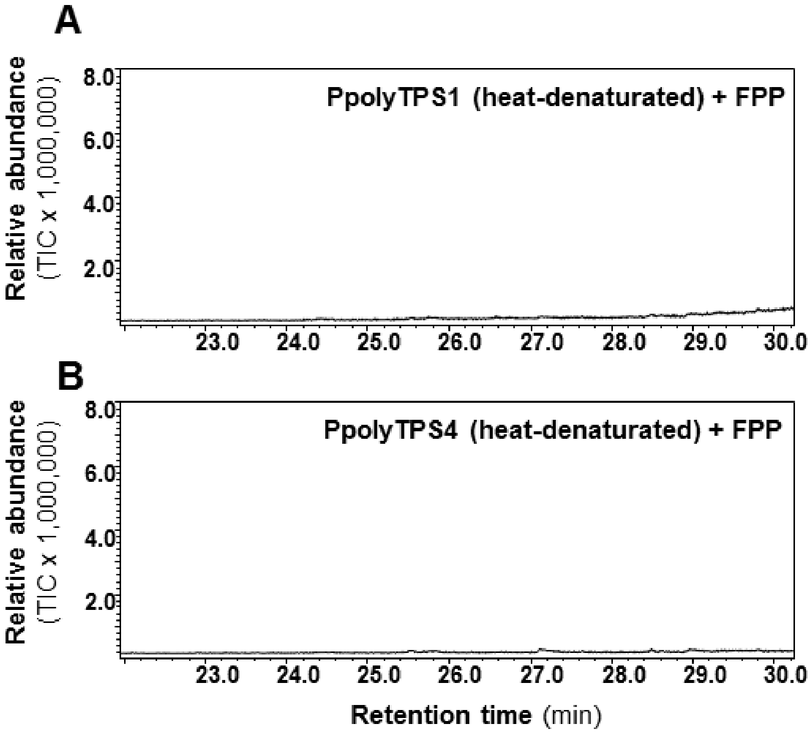

Figure S2. A. GC chromatogram of recombinant heat-denatured PpolyTPS1 assayed with farnesyl diphosphate (FPP). B. GC chromatogram of recombinant heat-denatured PpolyTPS4 assayed with FPP.
